# Allogeneic Hematopoietic Stem Cell Transplantation Achieves Durable Remission in Blast‐Phase Chronic Myeloid Leukemia Presenting as Early T‐Cell Precursor Acute Lymphoblastic Leukemia

**DOI:** 10.1002/jha2.70405

**Published:** 2026-09-23

**Authors:** Takafumi Tsushima, Yukiko Konishi, Natsumi Yoda, Koichiro Wada, Kento Okamoto, Kosuke Matsuo, Shuntaro Isogai, Sonoko Shimoji, Yoshikazu Utsu, Shin‐Ichi Masuda, Nobuyuki Aotsuka

**Affiliations:** ^1^ Department of Hematology and Oncology Japanese Red Cross Narita Hospital Narita Japan

## Abstract

**Introduction:**

The presentation of chronic myeloid leukemia (CML) in the blast phase as early T‐cell precursor acute lymphoblastic leukemia (ETP‐ALL) is very rare. As previously reported, in contrast to B‐ALL, the prognosis is poor.

**Case Presentation:**

We encountered a patient with blast phase CML presenting as ETP‐ALL. The patient responded to treatment with dasatinib and Hyper CVAD. However, the BCR::ABL1 transcript levels remained positive, and the patient demonstrated resistance to switching to ponatinib and other agents. Eventually, the patient underwent allogeneic hematopoietic stem cell transplantation (HSCT) and has maintained long‐term molecular remission.

**Conclusion:**

HSCT following remission induction therapy, inclusive of TKIs, may enhance prognosis for patients with blast phase CML presenting as ETP‐ALL.

**Trial Registration:**

The authors have confirmed clinical trial registration is not needed for this submission.

## 1 Introduction

The blast phase of chronic myeloid leukemia (CML) exhibits myeloid, lymphoid, and mixed phenotypes, with lymphoid phenotypes predominantly adopting the B‐cell acute lymphoblastic leukemia phenotype [1]. Early T‐cell precursor acute lymphoblastic leukemia (ETP‐ALL) is a distinct subtype of T‐lymphoblastic leukemia, characterized by its unique immunophenotypic and genomic profile [2]. The presentation of CML in the blast phase as ETP‐ALL has only been reported in a few cases and is very rare [3, 4, 5, 6]. In addition, BCR::ABL1 transcript positive ɤδ T‐cell leukemia has been reported [7]. There has been one case report of an allogeneic transplantation for extramedullary T‐ALL transformation of CML [8]. To date, only a single blast phase CML presenting as ETP‐ALL case of allogeneic transplantation has been reported [6]. There is a lack of comprehensive reports detailing the clinical trajectory leading to allogeneic hematopoietic stem cell transplantation (allo‐HSCT) in patients with CML presenting as ETP‐ALL, along with limited established treatment approaches. This report discusses a case of allo‐HSCT performed for CML in the blast phase presenting as ETP‐ALL.

## 2 Case Report

A 48‐year‐old male presented with the chief complaint of bilateral inguinal masses persisting for 1 month prior to admission. He also reported experiencing weight loss and night sweats. One year prior to admission, a routine health checkup revealed an elevated white blood cell count, but he did not seek further medical attention at that time. The exact white blood cell count at that time was unknown. On the day of admission, the patient consulted his primary care physician, and a complete blood count revealed leukocytosis, prompting a referral to our department. The patient's medical history included a traumatic subarachnoid hemorrhage at 24 years of age and acute epididymitis at 42 years of age. He was not taking any medications, and there was no family history of blood disorders.

The complete blood count results were abnormal (hemoglobin: 10.4 g/dL; white blood cell count: 102,100/µL; neutrophils: 39%; lymphocytes: 8%; blast cells: 16%; platelets: 288 × 10^3^/µL). Peripheral blood smears revealed large blast cells with a tendency to cluster. Laboratory findings were as follows: Lactate dehydrogenase: 598 U/L (normal range: 124–222 IU/L); total bilirubin: 0.5 mg/dL; C‐reactive protein: 0.56 mg/L; albumin: 4.3 g/dL; urea: 15 mg/dL; creatinine: 0.75 mg/dL. Serum β2‐microglobulin and soluble interleukin‐2 receptor (sIL‐2R) were elevated at 3.8 mg/L (normal range: 1.0–1.9 mg/L) and 1888.7 U/mL (normal range: 157–474 U/mL), respectively. The human T‐lymphotropic virus‐1/2 antibody test was negative. The major BCR::ABL1 mRNA/ABL mRNA international scale (IS) was positive at 84.2976%, and WT1‐mRNA was positive at 12,000 copies/µg RNA.

**FIGURE 1:**
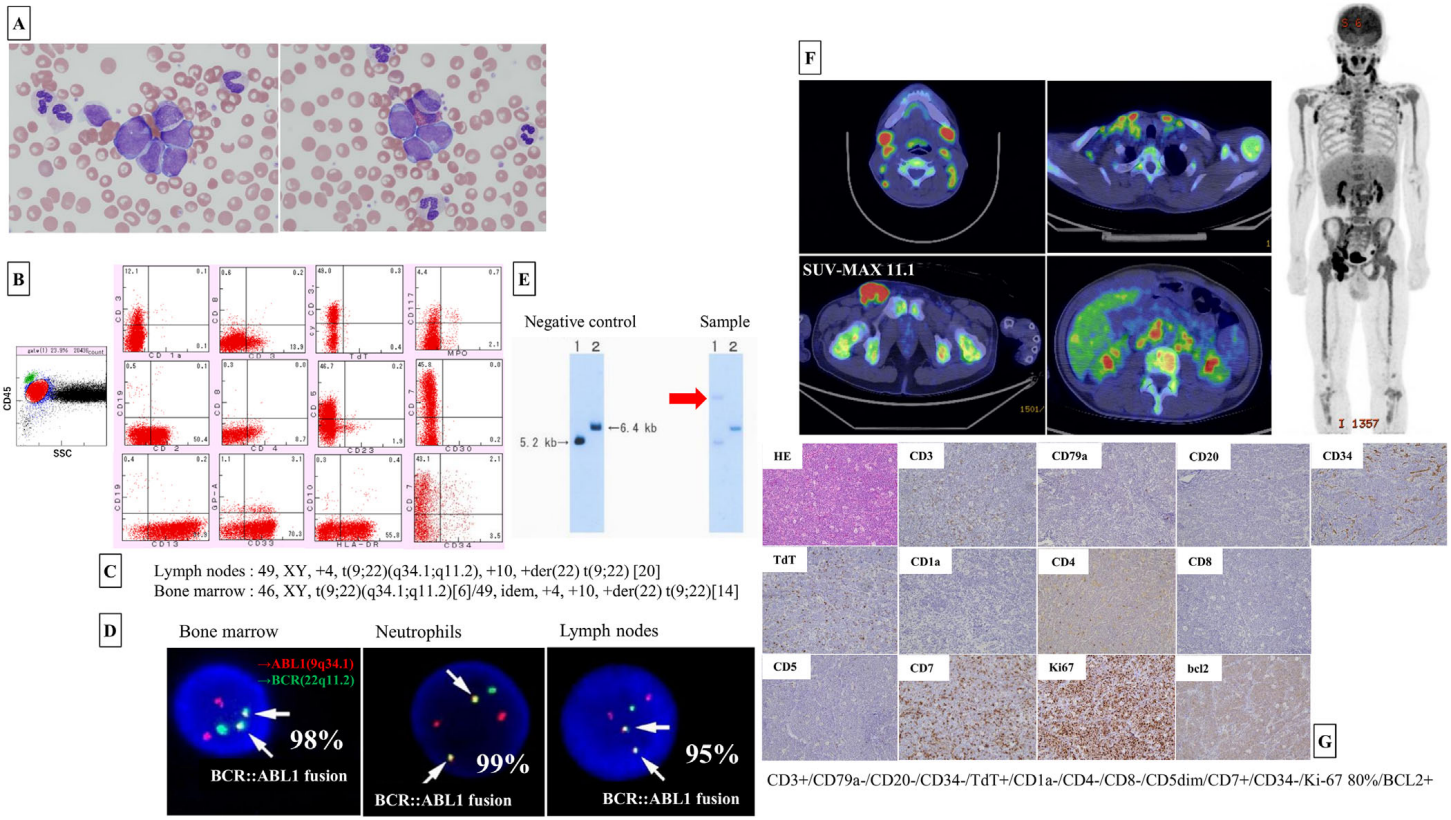
Diagnostic findings in blast‐phase CML presenting as ETP‐ALL. (A) Peripheral blood smear showing clusters of blast cells. (B) Flow cytometric analysis of bone marrow demonstrating a blast population expressing surface CD3^dim^, CD1a^−^, cytoplasmic CD3^+^, CD20^−^, CD4^−^, CD8^−^, CD5^dim^, CD2^+^, CD7^+^, CD13^+^, CD33^+^, HLA‐DR^+^, CD34^−^, and MPO^−^. (C) G‐banding karyotype analysis revealing a complex karyotype including *t*(9;22)(q34.1;q11.2). (D) Interphase fluorescence in situ hybridization demonstrating BCR::ABL1 fusion signals in bone marrow cells, neutrophils, and lymph node specimens. (E) Southern blot analysis confirming T‐cell receptor gene rearrangement. (F) Fluorodeoxyglucose‐positron emission tomography showing widespread lymphadenopathy. (G) Histopathologic examination of inguinal lymph node biopsy showing diffuse lymphocyte infiltration with immunohistochemical positivity for CD3, TdT, CD7, BCL2, and a high Ki‐67 proliferation index, and negativity for CD20, CD79a, CD34, CD1a, CD4, CD8, and CD5. BCL2: B‐cell lymphoma 2; MPO: myeloperoxidase; TdT: terminal deoxynucleotidyl transferase.

Bone marrow examination revealed an increase in blast cells, with clustering observed in 22.8% of the samples (Figure 1A). In terms of recurrent genetic abnormalities, major BCR::ABL chimerism was quantified as positive (58,000 copies/µg RNA). Flow cytometry revealed a phenotype of CD13^+^, CD33^+^, HLA‐DR^+^, myeloperoxidase^−^, TdT^−^, cytoplasmic CD3^+^, surface CD3^−^, CD1a^−^, CD8^−^, CD4^dim^, CD5^dim^, CD2^+^, and CD7^+^ (Figure 1B). Although there were no CD13/CD33 plots, Flow cytometry analyses such as CD13/CD19 and CD33/GP‐A showed that the tumor cells were CD13^+^ and CD33^+^. No NPM1 or FLT3‐ITD/TKD mutations were detected, and testing for other genetic mutations was not performed. G‐banding revealed a complex karyotype, including *t*(9;22)(q34.1;q11.2) (Figure 1C). Interphase fluorescence in situ hybridization (FISH) revealed a BCR::ABL1 fusion in peripheral blood neutrophils, bone marrow tumor cells, and lymphocytes from lymph node lesions (Figure 1D). Southern blot analysis confirmed T‐cell receptor (Jδ) gene rearrangement (Figure 1E). Fluorodeoxyglucose‐positron emission tomography revealed multiple lymphadenopathies in the cervical, axillary, inguinal, and para‐aortic regions, with a maximum standardized uptake value of 11.1 in the inguinal region (Figure 1F). Immunohistochemistry of the inguinal lymph nodes showed a profile of CD3^+^/CD79a^−^/CD20^−^/CD34^−^/TdT^+^/CD1a^−^/CD4^−^/CD8^−^/CD5^dim^/CD7^+^/CD34^−^/Ki‐67 80%/BCL2^+^ (Figure 1G). Immunohistochemistry for CD13 and CD33 was not performed. Regarding CD34 staining, while bone marrow vascular endothelial cells were positive, tumor cells were negative. Considering that CD3(cytoplasmic+/surface−), absent myeloperoxidase, absent CD1a and CD8, ≧ 25% of blasts with at least one of the myeloid markers (CD13, CD33, HLA‐DR), and dim to negative CD5, a diagnosis of ETP‐ALL was made in accordance with the World Health Organization Classification, fifth edition. Given the presence of BCR::ABL1 transcript positive findings in both neutrophils and lymphocytes, a final diagnosis of the blast phase of CML presenting as ETP‐ALL was established.

**FIGURE 2:**
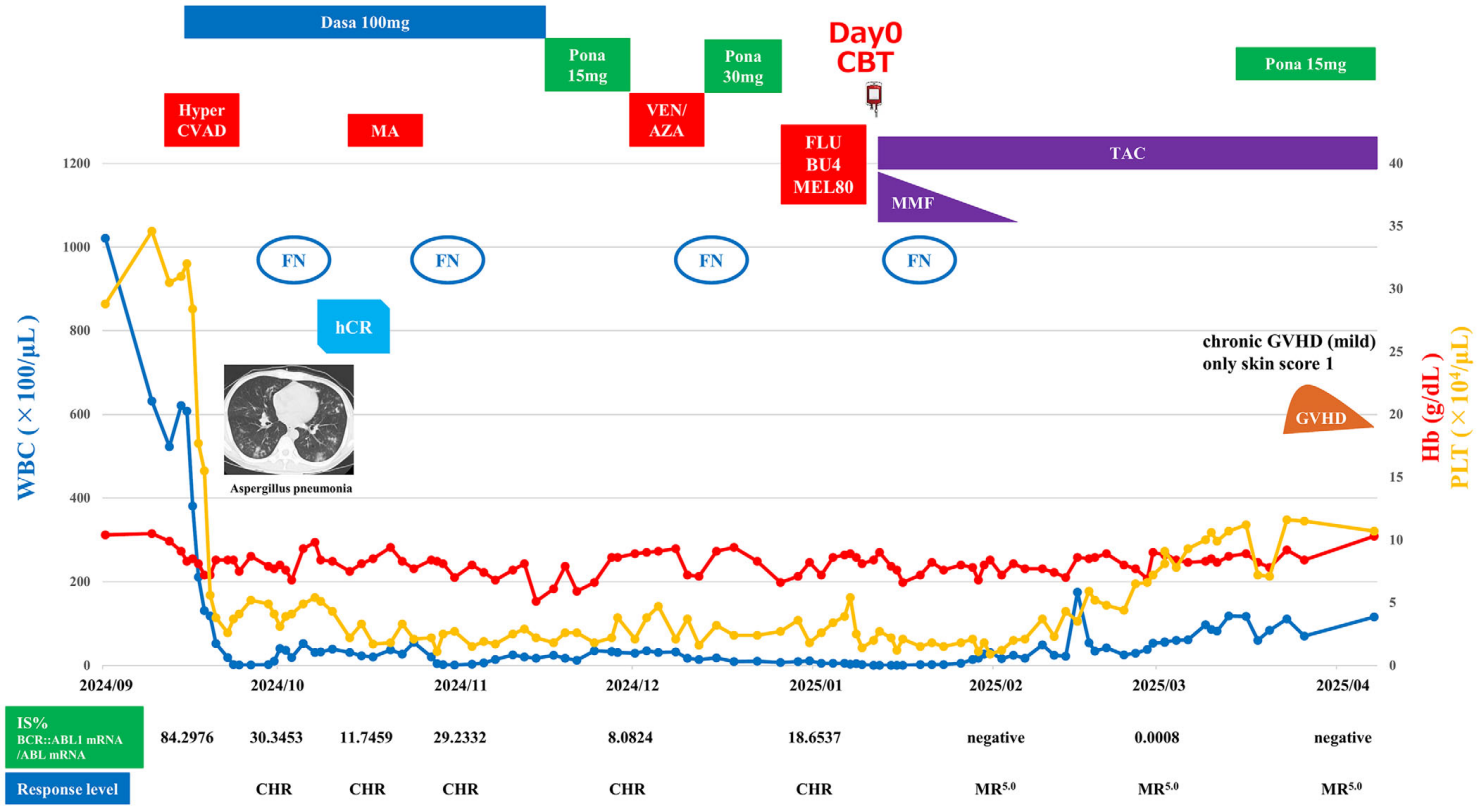
Clinical course and minimal residual disease dynamics during treatment. Timeline of treatment interventions and corresponding changes in minimal residual disease (MRD) assessed by BCR::ABL1/ABL international scale (IS), along with hematologic parameters. BU: busulfan; CBT: cord blood transplantation; CHR: complete hematologic response; Dasa: dasatinib; FN: febrile neutropenia; FLU: fludarabine; GVHD: graft‐versus‐host disease; Hb: hemoglobin; hCR: hematologic complete remission; Hyper‐CVAD: hyperfractionated cyclophosphamide, vincristine, doxorubicin, and dexamethasone; IS: International Scale; MA: high‐dose methotrexate plus cytarabine (Ara‐C); MEL: melphalan; MMF: mycophenolate mofetil; MR^5.0^: BCR::ABL1IS ≤ 0.001%; PLT: platelet; Pona: ponatinib; TAC: tacrolimus; VEM/AZA: venetoclax plus azacitidine.

The patient's clinical course is depicted in Figure 2. Initially, he received treatment consisting of a 100% dose of cyclophosphamide, vincristine, doxorubicin, and dexamethasone (HyperCVAD) alternating with high‐dose methotrexate and cytarabine (MA) in conjunction with dasatinib 100 mg/day. Although multiple lymphadenopathies resolved by the end of the first HyperCVAD cycle, The treatment response of BCR::ABL1 transcript remained at the level of complete hematologic response (CHR) (Figure 2). By the conclusion of MA therapy, the patient developed *Aspergillus pneumonia* and multiple episodes of febrile neutropenia. Despite the administration of dasatinib and HyperCVAD/MA, the treatment response remained at CHR, with no improvement (Figure 2), dasatinib was switched to ponatinib (15–30 mg/day), and treatment with venetoclax and azacitidine was initiated; however, there was still no improvement beyond a CHR in the treatment response even 3 months post‐diagnosis (Figure 2). In Japan, only azacitidine/venetoclax is approved for use in combination with venetoclax; since this case was positive for surface antigens such as CD13 and CD33, venetoclax plus azacitidine was selected as myeloid neoplasms. Intrathecal chemotherapy was administered on a monthly basis. At this point, there was a significant decline in hematopoiesis, and control of the underlying disease status remained poor. Considering previous reports indicating survival following allo‐HSCT [6], we opted to proceed with this procedure in this case.

To address the blast phase of CML presenting as ETP‐ALL, the patient underwent 4/6 allele‐matched cord blood transplantation with conditioning involving fludarabine (30 mg/m^2^ for 6 days, Days −7 to −2), busulfan (3.2 mg/kg for 4 days, Days −7 to −4), and melphalan (40 mg/m^2^ for 2 days, Days −3 to −2). A cord blood graft containing 1.09 × 10^5^ CD34 cells/kg was infused on Day 0. Graft‐versus‐host disease (GVHD) prophylaxis included tacrolimus and mycophenolate mofetil. Neutrophil engraftment was confirmed on Day 21, and the patient reached the MR^5.0^ level 1 month posttransplantation. Analysis of short tandem repeats in bone marrow samples using polymerase chain reaction confirmed that the bone marrow cells were in a complete chimeric state. Due to the absence of acute GVHD, mycophenolate mofetil was discontinued early (on Day 30) to enhance the graft‐versus‐leukemia effect. Given that engraftment of red blood cells and platelets was confirmed at 3 months posttransplant, maintenance therapy with 15 mg of ponatinib was initiated. Although mild chronic GVHD limited to the skin (Score 1) developed after starting ponatinib, it improved with topical corticosteroid treatment, and the patient has remained stable to date without the need for systemic corticosteroid therapy. The MR^5.0^ level was sustained continuously until 566 days after the transplantation.

## 3 Discussion

To the best of our knowledge, there are very few case reports of allo‐HSCT achieving minimal residual disease (MRD)‐negative status in the blast phase of CML presenting as ETP‐ALL. The lymphoid blast crisis phase constitutes nearly 30% of the blast phase in CML, with the B‐cell lineage being more prevalent [9]. In the chronic phase of CML, abnormalities other than the Philadelphia chromosome are infrequently observed, whereas additional chromosomal abnormalities are commonly detected in the accelerated or blast phases [10, 11]. In this case, multiple additional chromosomal abnormalities were evident from the initial visit. Neutrophil FISH is instrumental in differentiating between blast‐phase CML and de novo BCR::ABL1 transcript ‐positive ALL [12, 13]. This case was neutrophil FISH‐positive with a diagnosis of the blast phase of CML presenting as ETP‐ALL. If BCR::ABL1 transcript levels arises at the hematopoietic stem cell level, T‐lymphoblastic leukemia can develop within the T‐cell lineage [14]. In the context of the blast phase of CML presenting as ETP‐ALL, although tyrosine kinase inhibitors (TKIs) and chemotherapy demonstrate some therapeutic effect, their efficacy remains limited [3, 4, 5, 6]. One report confirmed long‐term survival following allo‐HSCT; however, that case exhibited residual MRD posttransplantation [6]. Based on previous reports, achieving long‐term remission through TKIs or chemotherapy may be challenging. The most curative approach is anticipated to involve performing allogeneic transplantation following tumor burden reduction with TKIs or chemotherapy. In this case, combination therapy with TKI and chemotherapy led to a rapid improvement in nodal lesions; however, BCR::ABL1 transcript levels remained significant, suggesting a heterogeneous disease process. The treatment course involved utilizing TKIs and chemotherapy to revert the ETP‐ALL to a state of CML alone, followed by allo‐HSCT.

In our case, hematopoietic stem cell transplantation following remission induction and consolidation therapy, including TKIs, successfully achieved MRD negativity. Venetoclax did not contribute significantly to the reduction of BCR::ABL1 transcript levels in this case, despite its benefits having been reported in ETP‐ALL [15]. While it continues to represent a treatment option for Philadelphia chromosome‐positive ETP‐ALL, several challenges remain to be addressed. The necessity of posttransplant TKI maintenance therapy in the blast phase of CML presenting as ETP‐ALL remains uncertain; however, given its poor prognosis, posttransplant TKI maintenance therapy should be considered.

In conclusion, there are few reports detailing the treatment course leading up to allogeneic transplantation in patients with CML in the blast phase presenting as ETP‐ALL. This case report offers a comprehensive account of the pathway to allo‐HSCT. Hematopoietic stem cell transplantation following remission induction therapy, inclusive of TKIs, may enhance prognosis for patients with blast phase CML presenting as ETP‐ALL.

## Author Contributions

T.T. designed the study, collected the data, and wrote the manuscript. All the authors have reviewed and approved the final manuscript.

## Funding

The authors have nothing to report.

## Ethics Statement

Ethical approval was not sought for the present study because it was only a single case report with the complete removal of all identifying information. The patient provided consent for the publication of this case, with the removal of all identifying information to maintain anonymity and privacy.

## Conflicts of Interest

The authors declare no conflicts of interest.

## Data Availability

The data supporting the findings of this study are available from the corresponding author.
